# Exophthalmia revealing acute myeloid leukemia

**DOI:** 10.11604/pamj.2025.50.14.46203

**Published:** 2025-01-07

**Authors:** Leila Debono, Youssef Jeddi

**Affiliations:** 1 Pediatric's Medical Emergency Department, Children's Hospital of Rabat, Rabat, Morocco,; 2 Faculty of Medicine and Pharmacy, Mohamed V University, Rabat, Morocco

**Keywords:** Exophthalmia, leukemia, child

## Image in medicine

A child aged 2 years and a half, without notable history, was presented with swelling of the right eye a month previously. He consulted a general practitioner, who put him on amoxicillin with clavulanic acid for 10 days and betamethasone drops for 5 days. A week later, there was swelling in the left eye. The parents consulted an ophthalmologist who treated him for conjunctivitis and put him on tobramycin eye drops for 5 days, without improvement. The clinical examination on admission found an irritable, afebrile, eupneic child with bilateral exophthalmos greater on the left with conjunctival redness and lower right eyelid ecchymosis. The rest of the examination was normal, notably free lymph node areas and no palpable mass. A cerebral and orbital Computed Tomography (CT) scan was performed, revealing Chandler's stage I preseptal cellulitis with bilateral grade I exophthalmos; the cerebral CT scan was without abnormalities. A blood count was performed revealing bicytopenia with normocytic normochromic anemia at 6.7 g/dl with thrombocytopenia at 19,000/µl, a white blood cell level at 25,600/µl with polynuclear neutrophils at a normal level and the presence of 66% blast cells. A smear carried out showed a blood cytological appearance in favor of acute myeloid leukemia.

**Figure 1:**
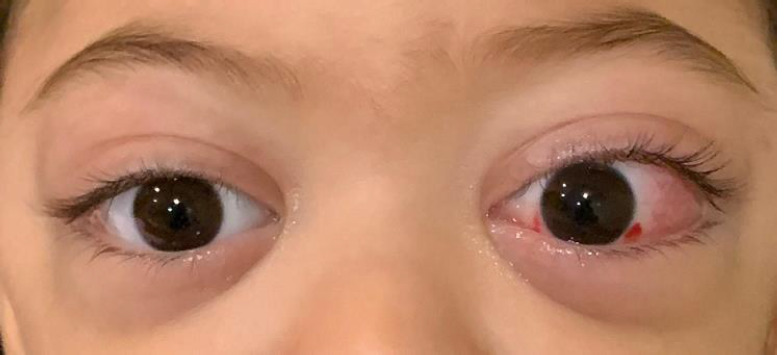
exophthalmia revealing acute myeloid leukemia

